# Instructional Videos for Students in Dental Medicine: Rules of Design and Correlations with Their Habits as Internet Consumers

**DOI:** 10.3390/ejihpe14060108

**Published:** 2024-06-05

**Authors:** Cristina Gena Dascalu, Claudiu Topoliceanu, Magda Ecaterina Antohe

**Affiliations:** 1 Department of Medical Informatics and Biostatistics, Faculty of Medicine, “Grigore T. Popa” University of Medicine and Pharmacy, 16 Universității Street, 700115 Iasi, Romania; 2 Department of Odontology, Periodontology, Fixed Restorations, Faculty of Dental Medicine, “Grigore T. Popa” University of Medicine and Pharmacy, 16 Universității Street, 700115 Iasi, Romania; claudiu.topoliceanu@umfiasi.ro; 3 Department of Implantology, Removable Dentures, Technology, Faculty of Dental Medicine, “Grigore T. Popa” University of Medicine and Pharmacy, 16 Universității Street, 700115 Iasi, Romania; magda.antohe@umfiasi.ro

**Keywords:** instructional videos, medicine students, dental students, e-learning, data clustering

## Abstract

Multimedia resources, such as instructional videos, are currently enjoying a certain popularity in the training programs for medical and dental students. The major challenge is to create such resources with quality content that is approved by students. In order to answer this challenge, it is imperative to find out which features of instructional videos are considered to be necessary and useful by students, thus being able to excite them, to hold their attention, and to stimulate them in learning with pleasure. Aim: We investigated the opinions of a sample of 551 students from four medical universities in Romania, in order to identify the students’ preferred characteristics in instructional videos, both globally and comparatively on genders and age groups and also according to their general preferences for using internet services. Material and methods: We used univariate (hypothesis testing) and multivariate (two-step clustering) data analysis techniques and revealed three clusters of students, primarily determined by their perceptions of the visual appearance of the instructional videos. Results: The structure of the clusters by gender and age group was relatively similar, but we recorded differences associated with the students’ expressed preferences for certain internet services compared to others. The first identified cluster (35.4% of the cases) contains students who prefer instructional videos to contain images used only for aesthetic purposes and to fill the gaps; they use internet services mainly for communication. The second cluster of students (34.8%) prefers videos designed as practical lessons, using explanatory drawings and diagrams drawn at the same time as the explanations; they also use internet services mainly for communication. The last cluster of students (29.8%) prefer videos designed as PowerPoint presentations, with animated pictures, diagrams, and drawings; they are slightly younger than the others and use internet services mainly for information and communication, but also for domestic facilities. Conclusions: The students’ preferences for certain features of instructional videos depend not only on gender and age but are also related to their developmental background and general opinions about modern technologies.

## 1. Introduction

A plethora of studies regarding the concept of “multimedia learning” has been conducted over time by specialists in education, psychology, computer science, linguistics, health care, and so on, with the first studies on this subject dating back to the 1930s [1,2]. In the early 1990s, Meyer and Anderson [3] demonstrated for the first time that people generally learn better from verbal and pictorial materials compared to only verbal materials; starting from this result, R.E. Meyer developed the first and best known theory on the effectiveness of multimedia learning techniques, namely the Cognitive Theory of Multimedia Learning (CTML), which is currently used as a benchmark by most researchers in the field [4,5,6,7]. Nowadays, the efforts of most researchers worldwide aim to improve the CTML theory in five main directions [8]: its theoretical foundations, its representations and principles, the instructional design and individual differences, associated motivations and metacognition, and the use of video and hypermedia, as well as to apply it practically in various educational fields, including the field of medical sciences [9,10] and for specific multimedia resources, with instructional videos being a relevant example among them [11,12]. In his theory, Meyer formulated 15 principles that must be followed in order to create successful educational materials [4], with success being defined by the author himself as “engaging the learner in appropriate cognitive processing during learning”, which guarantees the long-term acquisition of the taught notions. The validity of these principles has been verified not only by the author and his collaborators in more than 200 of their own experiments but also by many other experiments conducted by research teams around the world [13]. 

Our study belongs to this wide research direction, being focused on the investigation of the efficacy of instructional videos in medical education. Multimedia resources have recently revolutionized the style of teaching in undergraduate medical education, opening up a wide horizon of new and exciting opportunities. By enabling students to participate in immersive and dynamic learning sessions, this type of material allows them to understand complex concepts and develop real practical skills, thus acquiring the knowledge needed to become good professionals. Their integration into academic medical curricula offers a multitude of advantages, bringing a clear added value to the teaching process, with certain benefits for students, who receive a new and useful tool to prepare for real medical practice [14], with successful examples being reported in multiple fields [15,16,17,18,19]. Current technological progress allows teachers worldwide to create high-quality instructional videos at a low cost and time investment [20]. Although relatively easy to produce from a technical point of view, teaching materials of this type are very complex in structure, because they include most forms of digital content—real footage, animations, simulations, and interactive presentations. Therefore, certain skills are required to produce them, which refers to the ability to organize and structure the material in a form that is as clear, coherent, correct and, at the same time, attractive to the target audience as possible. In today’s online environment, there is a real influx of medical instructional videos (a general Google search using the keywords "medical procedure" and video resources yielded no less than 303,000,000 results), but their quality and content are very varied, so it is often difficult for teachers to identify the most competent materials, to choose the best method of using these materials and the optimal teaching framework in which to implement them, and possibly to decide whether it is better to use pre-existing videos or to create original content [21].

In spite of these dilemmas, instructional videos are still today an indispensable practical tool for modern education, with an undisputed position among digital tools, promoting certain advantages and benefits, among which we can mention the following:

- Stimulating student interest and autonomous, flexible learning based on visual stimuli.

Well-crafted instructional videos enable the creation of visual representations of complex medical concepts, facilitating the understanding of anatomical structures, physiological processes, surgical techniques, and clinical maneuvers. The visual learning provided by these materials promotes better understanding and retention of information [22,23]. Instructional videos are easy to distribute to students via educational websites and allow students to review material at their own pace and according to their own schedule, depending on their personal learning style [24,25,26].

- Realistic graphic simulations.

Instructional videos are one of the best suited tools to create realistic simulations of practical medical situations, allowing students to interact with virtual patients in interactive scenarios. They provide an immersive experience that facilitates the training of diagnostic skills, treatment planning, and clinical decision-making in a safe and controlled environment. A wide offering of simulators for training is currently available in medicine, ranging from static teaching videos to actors simulating human patients and full virtual reality (VR) systems, which represent the next level of advanced skills training, being both individual and collaborative [27,28,29].

- Access to advanced expert information.

Research studies show that video micro-lectures, delivered in real time or recorded in advance, are a successful pedagogical approach [21]. They are designed to complement traditional resources (textbooks, books, etc.) and to diversify the range of study options, simplifying access to teaching materials and providing in-depth, expert-level knowledge from professionals with extensive experience in the field [30], thus aligning with contemporary research [31]. This type of resource promotes health professionals’ freedom of opinion and expression; however, its strengths are equally weaknesses, because in the name of freedom of expression, material of questionable scientific value that is biased, incomplete, or even incorrect can just as easily be produced and promoted—and a censorship-type control is almost impossible to implement [32,33].

- Procedural demonstrations.

As we have already pointed out, in the medical field the main role of instructional videos is to illustrate complex procedures in detail, such as surgical techniques, patient assessments, or laboratory maneuvers [33]. However, in order to ensure the appropriate quality of such resources, Chauvet et al. [34] recommend, as far as possible, that they are standardized, receive regular updates, and are rapidly accessible through user-friendly platforms. There are also research groups that recommend the development of teaching videos based on CPA (cognitive process analysis) techniques, which provide students and residents with access to broader and deeper knowledge, allowing them to improve their analysis and clinical decision-making skills [35].

- Feedback and evaluation.

Feedback is an essential component in the educational process at any level, including undergraduate medical studies; its effectiveness, however, is determined by the nature, quantity, and mode of its delivery. Teaching videos may contain sections of assisted feedback, which are required for the training of the clinical skills of novice students and can be easily integrated in virtual OSCE (Objective Structured Clinical Examination) sessions [36,37,38,39,40].

- Interdisciplinary learning.

It is anticipated that interdisciplinary assessment will be essential in the near future to deliver high-quality care, even if nowadays it is rather difficult for universities to implement such educational programs related to clinical practice situations [41]. A simple solution is also brought by video resources [42], which can effectively illustrate interdisciplinary collaborations through real scenarios selected from practice, being the easiest way to convince students of the importance of teamwork and communication in medical practice.

It is, therefore, clear that instructional videos are one of the most effective learning tools, with multiple possibilities of use and indisputable benefits for both teachers and students. The major challenge, however, is to produce quality materials that are a real aid to the teaching process—not always a simple task as it requires an investment of time and resources and, equally importantly, the technical knowledge that university teachers do not necessarily have. 

There is strong evidence in the literature that creating valuable teaching videos requires not only specialist knowledge of the subject area but also a wide range of knowledge from other areas: IT, set design, directing, filming or audio recording techniques, and processing [21]. The optimal solution is, in fact, for universities to employ professionals from these related fields to work with the academic staff and to assist them during the production of video materials, but this obviously entails significant additional costs. In addition, studies have also shown that, apart from the technical aspects, quality teaching videos follow certain production principles that make them popular with students and enjoyable to watch, thus successfully achieving their intended purpose. 

The main purpose of our study was to investigate the validity of these principles, highlighted by the scientific literature, that make teaching videos popular in the particular case of medical and dental students. Our goal was to assess the extent to which such principles work in this category of students, who were investigated both globally and comparatively by their basic demographic features. We also aimed to refine the analysis by adding an additional working hypothesis, namely, that students’ specific preferences for certain characteristics of teaching videos are correlated with their general opinions and preferences for digital resources and modern technologies. Among the digital resources, we focused on internet services, which we divided into four main categories according to their purpose (information, communication, entertainment, and domestic facilities), and we studied comparatively the students’ preferences for certain characteristics of teaching videos in relation to the usual purpose for which they use internet services. This is an original approach, not encountered by us in the scientific literature, which allows the teacher to better understand the psychological profile of his students and their inner motivations, which lead them to agree or not with certain teaching tools. For maximum effect, instructional videos should be designed differently according to the type of students they are aimed at. 

## 2. Materials and Methods

Participants: In order to obtain a representative sample, between October 2022 and February 2023, we invited students in Medicine and Dental Medicine from all the main Romanian universities and all years of study to this study. We recorded the opinions of 551 students in Dental Medicine or General Medicine, belonging to all years of study, from four main universities in Romania (Iasi, Craiova, Timisoara, and Cluj-Napoca). The sample’s size was validated according to a calculation made for a finite population of 72,167 Romanian students enrolled in 2022–2023 in the faculties of Health and Social Assistance, with a confidence level of 95% and an accepted error of 5%. As a result, this study required a minimum of 383 participants.

Data collection: The students were asked to anonymously complete a 15-item online questionnaire in which they expressed their preferences for certain features that can be included in the instructional videos (Appendix A). The questionnaire also contained a supplementary question in which we proposed to the students four distinct categories of activities that can be carried out using internet services (information, communication, entertainment, and domestic facilities). We asked the students to rank these categories in order of preference (Appendix A). The questionnaire was presented and explained separately to each subject, along with the research goals. 

Questionnaire: The questionnaire was adapted and developed starting from the models already extant in the scientific literature [43,44,45,46]. Its validity and reliability were assessed using several methods (expert opinion, item analysis, the Kaiser–Meyer–Olkin coefficient, Bartlett’s test, and factor analysis); on these bases, the questionnaire was considered to be suitable for use in our study.

Variables: The study’s variables were the students’ answers to the questionnaire, which were analyzed comparatively by gender, age group, and preferences for using particular internet services.

Statistical analysis: The data from the questionnaire were recorded in a data file in SPSS 29.0 (SPSS Inc., Chicago, IL, USA) for Windows. The answers to each item were characterized through frequency distributions and contingency tables. The numerical variables were characterized through descriptive statistics (mean, standard deviation, range, and median). The comparisons between the samples were performed using the Chi-squared test for categorical data. We considered a *p* ≤ 0.05 value as statistically significant (*) and a *p* ≤ 0.01 value as statistically highly significant (**). In order to investigate the internal connections between the features of instructional videos, we used two-step clustering analysis, with the number of clusters being automatically calculated using Schwarz’s Bayesian Information Criterion (BIC) and a maximal limit of 15 clusters.

Ethical statement: Participation in our study was voluntary. The subjects were informed about this study and the content of the questionnaire, and they agreed with informed consent. The questionnaires were filled in anonymously, in order to protect the subjects’ privacy and to obtain objective answers as much as possible. This study was approved by the Ethical Committee of “Grigore T. Popa” University of Medicine and Pharmacy, Iasi, Romania.

## 3. Results

Three-quarters of the students were female (76.2%); most of the students were in years 1 and 2 of their studies (63.7%) and aged between 18 and 20 years old (53.9%), with a mean age of 21.78 ± 3.736 years; in total, 90% of the students had no previous higher education. The sample’s general features are presented in Table 1.

In general, there are not many significant differences between the genders in terms of their preferred features in instructional videos. However, it was observed that a higher proportion of males prefer the narrator to be male (61.1%), while females are almost equally divided between the two variants, although slightly more of them (51.4%) prefer the narrator to be female. Another statistically significant difference was noted in terms of the language in which the narrator speaks: the preferences are clearly towards Romanian. The majority of students want the text spoken by the narrator to be accompanied by subtitles at the bottom of the screen, but females who expressed this preference (75.7%) are significantly more numerous than males (64.9%). The majority of students want the spoken text to be accompanied by visuals highlighting the important concepts (95.5%). In addition, 55.0% of males prefer videos made as practical lessons, in which the teacher is recorded at the blackboard explaining and drawing, while 55.5% of females prefer the instructional videos to be in the form of a PowerPoint presentation with pictures, diagrams, and animated drawings (Table 2).

Furthermore, there are not many significant differences of opinion between the age groups. The younger students, aged 18–20, prefer the narrator to be male in 57.6% of cases, while 57.6% of those aged 21–24 and 52.1% of those over 25 prefer the narrator to be female. In general, students prefer the narrator to speak slowly, in percentages that increase as they get older, from 62.0% of 18–20 year olds to 77.8% of 21–24 year olds and 72.9% of over 25 year olds. The last element for which statistically significant differences between the three age groups was reported is the place of the explanatory texts accompanying the diagrams and drawings presented. The vast majority of students prefer to place them directly on the drawing in the area to which they refer; young students in particular, aged 18–20, express this preference (81.1%), and the percentage decreases as the students get older: 79.7% of students aged 21–24 and 64.6% of those over 25. Otherwise, no other statistically significant differences were noted (Table 3).

However, the objective of our study was to refine the obtained results by analyzing the basic demographic characteristics of the group through a more nuanced analysis of the preferences for the four general categories of internet services, because this analysis would provide useful information for the optimized design of instructional videos that are better adapted to the target audience.

We will detail the results obtained in this respect below. 

The students who use internet services primarily for information differ statistically significantly from the others in that more of them (48.9%) prefer instructional videos in which the teacher is recorded at the blackboard explaining and drawing. Other preferences that slightly, if not statistically significantly, stand out are as follows: the narrator should be female (50.0%), possibly speaking in English (23.9%), even at high speed (33.3%); it is better if the video does not contain images used only for aesthetic purposes to fill the gaps (52.2%); and the videos can be without subtitles at the bottom of the screen (30.6%). It is also found that, of the students who rank information as their second preferred internet service, a significantly higher percentage (16.4%) accept that the text spoken by the narrator is not accompanied by animated explanatory drawings (Table 4).

The students who use the internet mainly for communication are also those who prefer the narrator to speak in Romanian (86.2%). No other statistically significant differences were observed. Other preferences that highlight this category of students are that the narrator should still be female (50.3%), that the narrator should speak in a conversational and friendly style (70.4%), and that the film should not contain background music (73.1%). Explanatory texts should be placed directly on the drawing in the area to which it refers (79.9%), and already drawn diagrams that will only be explained in the video are acceptable (32.6%). It is also accepted that the video should present the subject matter heterogeneously in the form of free exposition (12.0%), but PowerPoint presentations with pictures, diagrams, and animated drawings are preferred (56.6%). In addition, a significant percentage of students in this category opt for videos with a maximum length of 6 min (25.1%) (Table 5).

The students who use internet services primarily for entertainment stand out statistically significantly from the other categories in that they prefer spoken text to be accompanied by subtitles at the bottom of the screen (78.1%), animated graphics to explain the text more clearly (90.2%), and the video to present the subject in a structured way that gradually moves from simple to complex (87.4%). A higher percentage of students in this category accept that the narrator speaks in English (21. 3%), but they prefer that the speaker maintains a low speaking speed (68.9%). The film may contain background music (32.2%). Explanatory texts should be placed directly on the drawing in the area to which they refer (82.0%). The students who rank entertainment last in their preferences stand out significantly from others in that they prefer to use already drawn explanatory drawings and diagrams, which will only be explained in the film (52.9%) (Table 6).

The students who use internet services for domestic facilities are not statistically significantly different from others in terms of their preferences for how instructional videos are put together. It can be noted, however, that it was in this category that the highest percentages of students were found to accept that the narrator speaks in English (25.7%) but at a slow speed (74.3%), that the film contains background music (34.3%) and images used only for aesthetic purposes to fill the gaps (51.4%), and that explanatory texts are placed at the bottom of the screen (28.6%) and diagrams and drawings are already drawn and only explained in the film (42.9%) A high percentage of students in this category also indicate that they prefer educational films with a length of 6 min or less (37.1%). In addition, students concerned with domestic facilities services, which are ranked second in the top preferences, prefer significantly more than the other categories that the narrator in instructional videos is female (61.8%) (Table 7).

**Figure 1:**
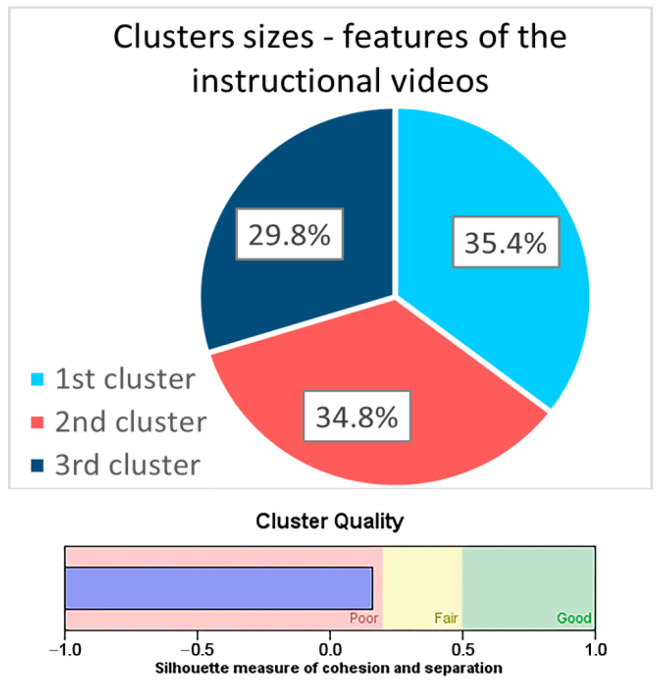
The sizes of the clusters identified among the preferred features of didactic videos.

**Figure 2:**
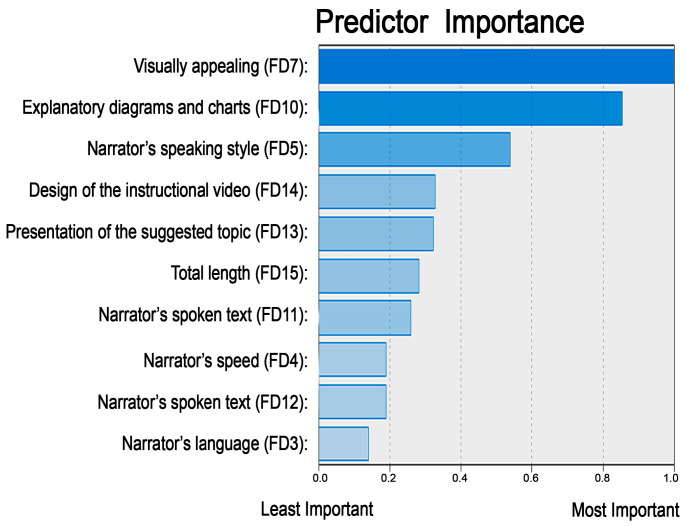
The classification of the 15 features according to their PI coefficients (predictor importance).

The classification of the 15 items of the characteristics of the instructional videos that were preferred by the students led to the identification of three clusters of approximately equal size (Figure 1). The most important predictor among the 15 proposed items was item FD7: visually appealing—images used only for aesthetic purposes vs. their absence (predictor importance PI = 1.00); followed by item FD10: explanatory drawings and diagrams already drawn vs. drawn simultaneously with explanations (PI = 0.85); and then item FD5: narrator’s conversational, friendly vs. formal, didactic style of expression (PI = 0.54) (Figure 2).

The classification of the students according to the agreed characteristics of instructional videos was therefore determined by the perception of their visual appearance. The first cluster identified (35.4% of the cases) contains the students who, in their vast majority, prefer instructional videos to contain images used only for aesthetic purposes, to fill the gaps (90.3%). These students also agree that the narrator’s voice should be female (59.5%) and that the narrator speaks in Romanian (94.4%), with a low speed (79.5%). All the students in this cluster want the narrator’s spoken text to be accompanied by animated diagrams to explain the text more clearly and by graphics to highlight the important concepts and for the subject to be presented in a structured way, explaining simple concepts first and then complex ones (96.9%). 

The second cluster also includes a third of the students (34.8%), but the vast majority of these students (88.0%) are not interested in instructional videos containing images used only for aesthetic purposes, to fill in the gaps, but want explanatory drawings and diagrams to be drawn at the same time as the explanations (94.8%). Other specific preferences for the students in this category are for the narrator’s voice to be male (65.6%), the spoken text to be accompanied by subtitles at the bottom of the screen (86.5%), and the explanatory text for diagrams and drawings to be displayed directly on them in the area to which they refer (89. 6%). The video should be designed as a practical lesson, with the teacher recorded at the blackboard explaining and drawing (65.1%), and should be as long as necessary, not requiring the 6 min rule (93.8%). 

The last cluster includes 29.8% of the students, who are also not interested in instructional videos containing images used only for aesthetic purposes, to fill the gaps (65.9%), but agree that the explanatory drawings and diagrams should be already drawn and only explained in the film (76.2%). Slightly more than half of the students in this category (52.4%) agree that the narrator should speak at a high speed, compared to no more than one-quarter of the students in the other clusters, who usually prefer the narrator to speak at a low speed. In addition, the vast majority of students in this cluster do not consider it necessary for the teaching films to contain background music (80.5%). Nearly one-third of the students agree that the films should also be made as free-form presentations (30.5%), and the vast majority (81.7%) want them to be designed as PowerPoint presentations with animated pictures, diagrams, and drawings (Table 8).

We also characterized the identified clusters according to the criteria we proposed in the other sections of this study: gender, age group, and the general preferences of the students among the four categories of internet services we defined. The results are presented in Table 9.

Overall, no statistically significant differences were identified. The three clusters have similar structures by genders and age groups, although it can still be observed that the third cluster comprises slightly more young students, aged 18–20 (59.1%), and the first two clusters comprise more students aged 21–24. The students’ preferences for the four categories of internet services tracked do not differ statistically significantly between the clusters separating them according to the preferred characteristics of the educational videos. The highest proportion of students using internet services for information is found in the third cluster (77.5%), and the highest proportion of students using internet services for communication is also found in the third cluster (91.4%), as well as in the first cluster (90.8%). The students using the internet mainly for entertainment are relatively evenly spread across the three clusters, and those using internet services for domestic facilities are clustered slightly more frequently in the third cluster (Table 9).

## 4. Discussion

The literature provides a wealth of data demonstrating the effectiveness of instructional videos in education in general and in university medical education in particular.

All these data are more or less related to the 15 principles of multimedia instructional design, formulated by R.E. Mayer in 2021 [4]:

“Principles for reducing extraneous processing:

1. Coherence principle: People learn better when extraneous material is excluded rather than included;
2. Signalling principle: People learn better when cues are added that highlight the organization of the essential material;
3. Redundancy principle: People do not learn better when printed text is added to graphics and narration; people learn better from graphics and narration than from graphics, narration, and printed text, when the lesson is fast-paced;
4. Spatial contiguity principle: People learn better when corresponding words and pictures are presented near rather than far from each other on the page or screen;
5. Temporal contiguity principle: People learn better when corresponding words and pictures are presented simultaneously rather than successively;

Principles for managing essential processing:

6. Segmenting principle: People learn better when a multimedia lesson is presented in user-paced segments rather than as a continuous unit;
7. Pretraining principle: People learn better from a multimedia lesson when they know the names and characteristics of the main concepts;
8. Modality principle: People learn better from graphics and narration than from graphics and on-screen text;

Principles for managing essential processing:

9. Multimedia principle: People learn better from words and pictures than from words alone;
10. Personalization principle: People learn better from multimedia lessons when words are in conversational style rather than formal style;
11. Voice principle: People learn better when the narration in multimedia lessons is spoken in a friendly human voice rather than a machine voice;
12. Image principle: People do not necessarily learn better from a multimedia lesson when the speaker’s image is added to the screen;
13. Embodiment principle: People learn more deeply from multimedia presentations when an onscreen instructor displays high embodiment rather than low embodiment;
14. Immersion principle: People do not necessarily learn better in 3D immersive virtual reality than with a corresponding 2D desktop presentation;
15. Generative activity principle: People learn better when they are guided in carrying out generative learning activities during learning”.

Our study allowed us to check the applicability of ten of these principles to the design of instructional videos with medical content, expressed through the students’ preferences for certain features in such didactic resources:

- Coherence principle: Most of our students are not interested in videos with background music (FD6, option 2: 71.5%) or images used only for aesthetic purposes to fill the gaps (FD7, option 2: 53.7%), without statistically significant differences between genders, age groups, and preferences for certain internet services. It has to be noted, however, that almost half of the students (46.3%) enjoy images used only for aesthetic purposes—especially males (49.6%), aged 21–24 years (49.4%), using the internet mainly for domestic facilities (51.4%).
- Signaling principle: Almost all students prefer the spoken text to be accompanied by visual elements highlighting the important elements (FD12, option 2: 95.5%). This feature is significantly more preferred by females (96.7%) than by males (91.6%).
- Redundancy principle: This was not verified in our study, because most students prefer videos accompanied by subtitles at the bottom of the screen (FD8, option 2: 73.1%). This feature is preferred significantly often by females (75.7%), using the internet mainly for entertainment (78.1%). However, this is not necessarily a contradiction, because our students also prefer slow-paced lessons, while the redundancy principle in Meyer’s vision regards fast-paced lessons.
- Spatial contiguity principle: Most of our students enjoy the explanatory text placed directly on the drawing in the area it refers to (FD9, option 1: 77.9%). This feature is preferred more often by females (78.8%), significantly younger—aged 18–20 years (81.1%)—also using the internet mainly for entertainment (82.0%).
- Temporal contiguity principle: Most of our students prefer the explanatory diagrams and charts to be drawn simultaneously with the explanations and not before (FD10, option 2: 67.3%). This feature is preferred more often by males (69.5%), aged 21–24 years (73.4%), using internet services mainly for communication (67.4%), even if statistically significant differences were not reported.
- Pretraining principle: Again, the students’ preferences are very clear: most of them prefer structured videos that first explain the basic notions and then the complex ones (FD13, option 2: 88.4%) without any significant differences between genders, age groups, and preferences for specific internet services. This feature is preferred more often by students aged 21–24 years (92.4%), who use internet services mostly for domestic facilities (91.4%) and rather rarely for entertainment (91.7%).
- Personalization principle: Our students enjoy clearly the conversational speaking style instead the formal style (FD5, option 1: 67.9%); the percentage of female students who enjoy this style is slightly higher than the corresponding percentage of males (68.3%). There are also high percentages for students over 25 years old (71.9%) and those who use internet services mainly for communication (70.4%).
- Voice principle: Almost all students prefer the human voice to be used in instructional videos (FD1, option 2: 98.4%) with almost no difference between genders, age groups, and preferences for certain internet services. Only among the students who use internet services mainly for domestic facilities is this percentage slightly lower (97.1%).
- Embodiment principle: This was only partially checked, because the students’ opinions are almost equally shared—53.0% of them prefer instructional videos designed as PowerPoint presentations, while the other 47.0% of them prefer instructional videos where the teacher is recorded at the blackboard, where he explains and draws, therefore showing a higher embodiment (FD14, option 1). This option is significantly more popular among male students compared with female (55.0% vs. 44.5%), as well as among the students between 21 and 24 years old (50.0%), who use internet services mainly for information (48.9%).

The other five principles of Mayer were not covered by the items contained in our questionnaire, a fact that could be classified as a possible limitation of our study (although it can easily be solved in the future by redesigning the questionnaire we used). Another limitation in the same regard is that the efficacy of Mayer’s principles was only verified indirectly, by investigating the students’ opinions about the principles, and not directly, by checking the students’ knowledge level before and after using the instructional videos designed according to the principles.

Such limitations were reported in the scientific literature as well. Usually, the CTML principles are inconsistently applied [47], with the instructional videos most often failing to follow the principles of coherence, segmenting, and signaling [48]. This is why practical guides have been proposed, in order to describe in-depth the CTML and the required steps to apply it in the design of instructional videos [11]. The newest approach is to use Artificial Intelligence (AI) tools in (re)designing instructional videos tailored according to the CTML principles [12]; such tools have a great potential to generate high-quality content and therefore to significantly enhance the learning experience.

The scientific literature also provides a wide range of practical results attesting the effectiveness of multimedia learning tools in general and particularly of teaching videos. Some relevant examples are discussed below, with direct references to university medical education. 

Leela et al. [38] conducted a cross-sectional study of 324 third- and fourth-year medical and dental students in a private university; the students were interviewed about their preferences for teaching methods. It was found that the teaching tool most preferred by the students was instructional videos (75.1%), followed by question and answer-oriented learning (70.0%), problem-oriented learning (69.6%), interactive discussions (69.3%), case studies (67.2%), traditional lectures (66.0%), and PowerPoint presentations (65.9%). Dental students ranked video content first in order of preference, followed by PowerPoint presentations and traditional lectures, while medical students preferred interactive discussions, followed by quizzes and debates. This research group therefore shows that there is a need to move away from the traditional teaching style to a combination of alternative methods, including video resources, problem-based learning (PBL), and interactive discussions, which motivate students much better and are, thus, significantly more effective. 

Ortega et al. [31] developed in 2017 the first textbook combined with interactive video content dedicated to postgraduate medical training and investigated its effectiveness through a questionnaire proposed to the residents who used it. The results of the survey conducted again indicate the preference of medical residents for learning environments that integrate technology, combining traditional textbooks with interactive multimedia elements. Miranda et al. [49] produced narrative videos to illustrate neurosurgical maneuvers, which they integrated into a telehealth system used for the distance training of students during the COVID-19 pandemic; all students monitored used these resources to learn, and the vast majority found them extremely useful for understanding the concepts presented. Doherty et al. [22] assessed the correlation between students’ level of engagement in using lecture-style instructional videos and various characteristics of the videos. The videos tested presented topics in anatomy, physiology, and clinical assessment, and student engagement was quantified by the amount of viewing time they allocated. It was found that the most viewed videos were those divided into relatively short segments, without subtitles and highlighting important information through different graphic elements. The students also perceived positively the appearance in the video, at regular intervals, of a human instructor to provide explanations. When a lesson consisted of several videos, the research group recommended that the most important concepts were discussed first in the sequence.

Chauvet et al. [34] also studied the characteristics of effective instructional videos, in the particular case of describing surgical maneuvers. They too showed that the duration of the videos should be relatively short, 10 to 15 min, while also highlighting a number of specific elements: it was recommended that the videos provide details about the patient’s preparation for the procedure (e.g., his or her body mass index, the degree of the Trendelenburg position, or the pneumoperitoneum pressure), that they are organized in distinct stages, and that they are uploaded to both educational and open platforms, where they should remain fully accessible to those interested. It was further recommended that the videos show not only the correct maneuvers but also the mistakes that can be made (such as ‘so no’), because this approach is particularly appreciated by young surgeons.

Our data also confirm that students are very positive about instructional videos. As regards the recommended features for the design of the videos, in some cases we recorded different opinions. Thus, three-quarters of students (74.6%) do not particularly prefer shorter videos, with no significant differences between genders or age groups. Three-quarters of students (73.1%) also prefer videos with subtitles—mostly females, regardless of age. Almost all students (95.5%) want videos to contain graphics to highlight important concepts, and opinions are almost evenly divided on the presence of the human instructor: 47.0% of students like videos designed as lectures, where the teacher is filmed at the blackboard, while 53.0% like videos designed as PowerPoint presentations, with pictures and diagrams. 

Other, more general issues related to the effectiveness of teaching medical videos as part of more complex tools were also investigated.

Thus, Palter and Grantcharov [50] investigated whether one-on-one practice on virtual reality (VR) simulators that include video sequences leads to improved surgical skills. They demonstrated that the resident physicians who trained on VR simulators developed superior skills compared to those who did not use such systems, even though their initial skill level was similar. 

Melkers et al. [30] were concerned with the effectiveness of video microlectures in dentistry. They analysed 89 blogs and podcasts designed to provide professional content in various dental fields. 55% of their authors were practising dentists or hygienists, 30% were consultants, 9% were editors and moderators of discussion forums, and the rest were representatives of various professional organisations or corporations. The study authors thus highlighted the involvement of dentists and practicing hygienists in using social media platforms to disseminate valuable clinical information to dental colleagues and dental students. Lin et al. [32] and Paterson et al. [33], respectively, studied the quality benchmarks of video materials disseminated in medical educational blogs and podcasts to quantify the credibility and validity of this type of resource as a viable tool for medical education. A short list of quality indicators, significant for assessing credibility and relevance, has thus been highlighted: clear indication of authors and their expertise in the field, bibliographical references and possible conflicts of interest; explicit differentiation between personal opinions and factual statements, and respectively between the content itself and advertising material; delivery of accurate and professional information, consistent with the bibliographical references used and relevant to the intended audience; technical compatibility of the material with standard equipment, ensuring quick, easy and smooth access.

Matthan et al. [36] explored the side effects of video feedback, namely the potential anxiety induced in students when they perform simulated practical procedures. Two groups of students were comparatively analyzed: one group that received supervied individualized video feedback (SIVF) and the other group that received general, unsupervised video feedback (GUVF). The majority of subjects reported that neither type of feedback induced significant stress for them, although they preferred individualized feedback (SIVF).

Rammell et al. [40] investigated the extent to which students retained practiced practical skills over a 7-week follow-up period after receiving either synchronous video feedback from experts or unsupervised asynchronous video feedback. The students who received synchronous video feedback from experts demonstrated significant improvement in practiced skills throughout the study period, whereas no significant change was observed in the others. Seifert et al. [37] also compared the effectiveness of individualized but unsupervised video feedback compared to conventional, direct, expert feedback in learning practical oral and maxillofacial surgery skills. The research group concluded that while direct feedback from experts is the most effective method, other feedback options are also viable alternatives. On the other hand, Phillips et al. [39] did not report significant additional benefits of direct expert feedback, because similar results can be obtained using general videos made by experts, based on which students self-assess their own performance. We did not include this issue in our study, but it is exciting and may be a direction for further research.

Nisbet et al. [42,51] and Jorm et al. [41] addressed the issue of interdisciplinary learning strategies using video tools. The research group initiated an experiment in which students with different medical specialties were grouped into interdisciplinary teams and asked to produce a treatment plan for a proposed clinical scenario. The treatment plan was to be presented in the form of a 5 min video and a one-page written summary, after which the student teams were asked to peer review three other videos made by other teams participating in the experiment, based on predefined criteria: the correctness of the proposed treatment plan, the quality of the interdisciplinary collaboration, and the effectiveness of the video tools used to capture the audience’s attention. It was shown that the tasks proposed to the students—to present the treatment plan in the form of a video and to evaluate the work of their peers—stimulated their creativity and led to effective collaboration between them, thus being effective as an interdisciplinary learning strategy. The feedback from the participating students was also positive: 70.0% of them found the proposed tasks useful and 87.0% appreciated the relevance of their case study. The students also appreciated the contributions of their teammates and the importance of collaboration between them for the correct analysis of the clinical case and found the requirement to present their work in the form of a video innovative and stimulating.

The new approach we have proposed belongs to these attempts to better understand and optimize the efficacy of such tools. We focused on a deepened investigation of the reasons that students prefer or not certain features of instructional videos. In our opinion, such reasons cannot be related only to basic demographic features, but they have to be related as well to the students’ personalities, general preferences, and developmental background. We did indeed find significant results in this direction, which can be further investigated by focusing more on the students’ psychological features. Another novel approach was to classify students according to the features they preferred in the instructional videos by automatic clustering using the two-step clustering procedure. We chose this method of data analysis, because in this way we were able to not only identify the relevant clusters among the students but also the most important predictors responsible for their creation. The predictors that we found are interesting to analyze because they are not similar to the items initially reported as most popular. This approach is original, because the literature consulted indicates that it has not been used before in studies regarding students’ views on different features of the teaching process. Most studies of this type simply record student responses and compare them according to different criteria, without investigating the structure of the items. The analysis of the items using classification techniques, such as the one we have chosen, is able to provide more details, allowing the prioritization and grouping of the students according to certain hierarchies. In this way, we can better understand the intimate reasons why students do or do not prefer a teaching tool and, consequently, we can make an informed selection of the optimal methods to make it effective.

The possible limitations of our study derive from the investigated sample’s structure and from this study’s general design. Thus, even if the sample’s size was consistent, the biggest number of students belongs to the “Grigore T. Popa” University of Medicine and Pharmacy, Iasi, Romania, in the Faculty of Dental Medicine. Much more interesting results could be obtained by investigating a larger number of students from other medical faculties (general medicine or pharmacy) from all the other universities in the country. The questionnaire’s validation through Cronbach’s alpha coefficient of reliability gave us only a value of 0.200, which is rather poor. This is a not surprising fact since the items in the questionnaire are not recorded on the Likert scale and they investigate different features of the instructional videos, which are addressed to the quality of such material and are not necessarily related. However, this is also an aspect that can be fixed. The study’s design was standard: we focused on the answers of the students who voluntarily agreed to participate in this study, without investigating the opinions of the non-respondents through follow-up interviews. This could be a possible source of bias, but the questionnaire was filled in anonymously online, so it did not allow further investigations. Neverthless, we intend to continue this study through follow-up interviews on a smaller sample of respondents by comparison with a similar sample of non-respondents, in order to obtain a more finely-grained view of the results. 

## 5. Conclusions

Our study confirms the data in the literature, according to which students highly approve of instructional videos, although in some cases we recorded different opinions regarding the recommended characteristics for designing this type of material.

We confirmed the aim of our study, because we indeed found that the students’ preferences for certain features of instructional videos not only depend on gender and age but are also related to their developmental background and general opinions about modern technologies. The technologically savvy students, who are attracted to gadgets, computers, and the internet, also tend to be more open to all modern learning technologies and, in particular, to instructional videos, and we indeed found significant results and definite associations between these elements, as already described.

The obtained results are suggestive and paint a different, more complex picture, in our opinion, of both the digital teaching tools that can work in higher medical education and the typology of students who will enjoy using such tools and be able to make the most of their benefits.

## Figures and Tables

**Table 1:** General features of the study group.

		*n*	%
Gender	male	131	23.8
	female	420	76.2
Age group	18–20 years	297	53.9
	21–24 years	158	28.7
	over 25 years	96	17.4
University	UMF “Grigore T. Popa”, Iași	356	64.6
	UMF, Craiova	108	19.6
	UMF “Victor Babeș”, Timișoara	80	14.5
	UMF “Iuliu Hațieganu”, Cluj-Napoca	7	1.3
Previously graduated university studies	yes	55	10.0
	no	496	90.0
Total		551	100.0

**Table 2:** Features preferred by students in instructional videos—comparative study of genders.

	Gender				Total		*p*-Value
	Male		Female				
	Option 1: *n* (%)	Option 2: *n* (%)	Option 1: *n* (%)	Option 2: *n* (%)	Option 1: *n* (%)	Option 2: *n* (%)	
FD1	2 (1.5)	129 (98.5)	7 (1.7)	413 (98.3)	9 (1.6)	542 (98.4)	0.912
FD2	51 (38.9)	80 (61.1)	216 (51.4)	204 (48.6)	267 (48.5)	284 (51.5)	0.012 *
FD3	37 (28.2)	94 (71.8)	67 (16.0)	353 (84.0)	104 (18.9)	447 (81.1)	0.002 **
FD4	39 (29.8)	92 (70.2)	135 (32.1)	285 (67.9)	174 (31.6)	377 (68.4)	0.610
FD5	87 (66.4)	44 (33.6)	287 (68.3)	133 (31.7)	374 (67.9)	177 (32.1)	0.681
FD6	38 (29.0)	93 (71.0)	119 (28.3)	301 (71.7)	157 (28.5)	394 (71.5)	0.881
FD7	65 (49.6)	66 (50.4)	190 (45.2)	230 (54.8)	255 (46.3)	296 (53.7)	0.380
FD8	46 (35.1)	85 (64.9)	102 (24.3)	318 (75.7)	148 (26.9)	403 (73.1)	0.015 *
FD9	98 (74.8)	33 (25.2)	331 (78.8)	89 (2.2)	429 (77.9)	122 (22.1)	0.336
FD10	40 (30.5)	91 (69.5)	140 (33.3)	280 (66.7)	180 (32.7)	371 (67.3)	0.551
FD11	15 (11.5)	116 (88.5)	37 (8.8)	383 (91.2)	52 (9.4)	499 (90.6)	0.367
FD12	11 (8.4)	120 (91.6)	14 (3.3)	406 (96.7)	25 (4.5)	526 (95.5)	0.015 *
FD13	15 (11.5)	116 (88.5)	49 (11.7)	371 (88.3)	64 (11.6)	487 (88.4)	0.946
FD14	72 (55.0)	59 (45.0)	187 (44.5)	233 (55.5)	259 (47.0)	292 (53.0)	0.037 *
FD15	32 (24.4)	99 (75.6)	108 (25.7)	312 (74.3)	140 (25.4)	411 (7.6)	0.768
Total	131 (100.0)		420 (100.0)		551 (100.0)		

Pearson Chi-squared test; * *p* < 0.05 statistically significant; and ** *p* < 0.01 highly statistically significant.

**Table 3:** Features preferred by students in instructional videos—comparative study of age groups.

	Age Group						Total		*p*-Value
	18–20 Years		21–24 Years		Over 25 Years				
	Option 1: *n* (%)	Option 2: *n* (%)	Option 1: *n* (%)	Option 2: *n* (%)	Option 1: *n* (%)	Option 2: *n* (%)	Option 1: *n* (%)	Option 2: *n* (%)	
FD1	6 (2.0)	291 (98.0)	2 (1.3)	156 (98.7)	1 (1.0)	95 (99.0)	9 (1.6)	542 (98.4)	0.734
FD2	126 (42.4)	171 (57.6)	91 (57.6)	67 (42.4)	50 (52.1)	46 (47.9)	267 (48.5)	284 (51.5)	0.006 **
FD3	57 (19.2)	240 (80.8)	33 (20.9)	125 (79.1)	14 (14.6)	82 (85.4)	104 (18.9)	447 (81.1)	0.451
FD4	113 (38.0)	184 (62.0)	35 (22.2)	123 (77.8)	26 (27.1)	70 (72.9)	174 (31.6)	377 (68.4)	0.001 **
FD5	203 (68.4)	94 (31.6)	102 (64.6)	56 (35.4)	69 (71.9)	27 (28.1)	374 (67.9)	177 (32.1)	0.465
FD6	80 (26.9)	217 (73.1)	55 (34.8)	103 (65.2)	22 (22.9)	74 (77.1)	157 (28.5)	394 (71.5)	0.086
FD7	136 (45.8)	161 (54.2)	78 (49.4)	80 (50.6)	41 (42.7)	55 (57.3)	255 (46.3)	296 (53.7)	0.569
FD8	86 (29.0)	211 (71.0)	38 (24.1)	120 (75.9)	24 (25.0)	72 (75.0)	148 (26.9)	403 (73.1)	0.480
FD9	241 (81.1)	56 (18.9)	126 (79.7)	32 (20.3)	62 (64.6)	34 (35.4)	429 (77.9)	122 (22.1)	0.002 **
FD10	107 (36.0)	190 (64.0)	42 (26.6)	116 (73.4)	31 (32.3)	65 (67.7)	180 (32.7)	371 (67.3)	0.123
FD11	28 (9.4)	269 (90.6)	13 (8.2)	145 (91.8)	11 (11.5)	85 (88.5)	52 (9.4)	499 (90.6)	0.694
FD12	16 (5.4)	281 (94.6)	7 (4.4)	151 (95.6)	2 (2.1)	94 (97.9)	25 (4.5)	526 (95.5)	0.400
FD13	42 (14.1)	255 (85.9)	12 (7.6)	146 (92.4)	10 (10.4)	86 (89.6)	64 (11.6)	487 (88.4)	0.107
FD14	133 (44.8)	164 (55.2)	79 (50.0)	79 (50.0)	47 (49.0)	49 (51.0)	259 (47.0)	292 (53.0)	0.521
FD15	66 (22.2)	231 (77.8)	51 (32.3)	107 (67.7)	23 (24.0)	73 (76.0)	140 (25.4)	411 (7.6)	0.060
Total	297 (100.0)		158 (100.0)		96 (100.0)		551 (100.0)		

Pearson Chi-squared test; ** *p* < 0.01 highly statistically significant.

**Table 4:** Features preferred by students in instructional videos—comparative study on preferred internet services: information.

	Main Activity on Internet: Information								*p*-Value
	The Least Important		Less Important		Important		The Most Important		
	Option 1: *n* (%)	Option 2: *n* (%)	Option 1: *n* (%)	Option 2: *n* (%)	Option 1: *n* (%)	Option 2: *n* (%)	Option 1: *n* (%)	Option 2: *n* (%)	
FD1	-	19 (100.0)	5 (3.6)	133 (96.4)	2 (0.9)	212 (99.1)	2 (1.1)	178 (98.9)	0.197
FD2	9 (47.4)	10 (52.6)	62 (44.9)	76 (55.1)	106 (49.5)	108 (50.5)	90 (50.0)	90 (50.0)	0.809
FD3	5 (26.3)	14 (73.7)	24 (17.4)	114 (82.6)	32 (15.0)	182 (85.0)	43 (23.9)	137 (76.1)	0.112
FD4	8 (42.1)	11 (57.9)	37 (26.8)	101 (73.2)	69 (32.2)	145 (67.8)	60 (33.3)	120 (66.7)	0.436
FD5	11 (57.9)	8 (42.1)	94 (68.1)	44 (31.9)	151 (70.6)	63 (29.4)	118 (65.6)	62 (34.4)	0.568
FD6	3 (15.8)	16 (84.2)	47 (34.1)	91 (65.9)	55 (25.7)	159 (74.3)	52 (28.9)	128 (71.1)	0.218
FD7	10 (52.6)	9 (47.4)	63 (45.7)	75 (54.3)	96 (44.9)	118 (55.1)	86 (47.8)	94 (52.2)	0.881
FD8	6 (31.6)	13 (68.4)	31 (22.5)	107 (77.5)	56 (26.2)	158 (73.8)	55 (30.6)	125 (69.4)	0.411
FD9	17 (89.5)	2 (10.5)	102 (73.9)	36 (26.1)	170 (79.4)	44 (20.6)	140 (77.8)	40 (22.2)	0.385
FD10	10 (52.6)	9 (47.4)	41 (29.7)	97 (70.3)	69 (32.2)	145 (67.8)	60 (33.3)	120 (66.7)	0.257
FD11	1 (5.3)	18 (94.7)	3 (2.2)	135 (97.8)	35 (16.4)	179 (83.6)	13 (7.2)	167 (92.8)	0.000 **
FD12	1 (5.3)	18 (94.7)	3 (2.2)	135 (97.8)	16 (7.5)	198 (92.5)	5 (2.8)	175 (97.2)	0.061
FD13	1 (5.3)	18 (94.7)	15 (10.9)	123 (89.1)	29 (13.6)	185 (86.4)	19 (10.6)	161 (89.4)	0.615
FD14	9 (47.4)	10 (52.6)	76 (55.1)	62 (44.9)	86 (40.2)	128 (59.8)	88 (48.9)	92 (51.1)	0.049 *
FD15	5 (26.3)	14 (73.7)	39 (28.3)	99 (71.7)	50 (23.4)	164 (76.6)	46 (25.6)	134 (74.4)	0.783
Total	19 (100.0)		138 (100.0)		214 (100.0)		180 (100.0)		

Pearson Chi-squared test; * *p* < 0.05 statistically significant; and ** *p* < 0.01 highly statistically significant.

**Table 5:** Features preferred by students in instructional videos—comparative study on preferred internet services: communication.

	Main Activity on Internet: Communication								*p*-Value
	The Least Important		Less Important		Important		The Most Important		
	Option 1: *n* (%)	Option 2: *n* (%)	Option 1: *n* (%)	Option 2: *n* (%)	Option 1: *n* (%)	Option 2: *n* (%)	Option 1: *n* (%)	Option 2: *n* (%)	
FD1	-	5 (100.0)	-	58 (100.0)	3 (1.9)	151 (98.1)	6 (1.8)	328 (98.2)	0.754
FD2	1 (20.0)	4 (80.0)	20 (34.5)	38 (65.5)	78 (50.6)	76 (49.4)	168 (50.3)	166 (49.7)	0.075
FD3	3 (60.0)	2 (40.0)	22 (37.9)	36 (62.1)	33 (21.4)	121 (78.6)	46 (13.8)	288 (86.2)	0.000 **
FD4	1 (20.0)	4 (80.0)	18 (31.0)	40 (69.0)	51 (33.1)	103 (66.9)	104 (31.1)	230 (68.9)	0.915
FD5	2 (40.0)	3 (60.0)	36 (62.1)	22 (37.9)	101 (65.6)	53 (34.4)	235 (70.4)	99 (29.6)	0.262
FD6	2 (40.0)	3 (60.0)	18 (31.0)	40 (69.0)	47 (30.5)	107 (69.5)	90 (26.9)	244 (73.1)	0.750
FD7	1 (20.0)	4 (80.0)	26 (44.8)	32 (55.2)	71 (46.1)	83 (53.9)	157 (47.0)	177 (53.0)	0.680
FD8	-	5 (100.0)	15 (25.9)	43 (74.1)	46 (29.9)	108 (70.1)	87 (26.0)	247 (74.0)	0.442
FD9	3 (60.0)	2 (40.0)	44 (75.9)	14 (24.1)	115 (74.7)	39 (25.3)	267 (79.9)	67 (20.1)	0.423
FD10	-	5 (100.0)	15 (25.9)	43 (74.1)	56 (36.4)	98 (63.6)	109 (32.6)	225 (67.4)	0.203
FD11	-	5 (100.0)	7 (12.1)	51 (87.9)	14 (9.1)	140 (90.9)	31 (9.3)	303 (90.7)	0.796
FD12	-	5 (100.0)	5 (8.6)	53 (91.4)	6 (3.9)	148 (96.1)	14 (4.2)	320 (95.8)	0.439
FD13	-	5 (100.0)	5 (8.6)	53 (91.4)	19 (12.3)	135 (87.7)	40 (12.0)	294 (88.0)	0.733
FD14	3 (60.0)	2 (40.0)	31 (53.4)	27 (46.6)	80 (51.9)	74 (48.1)	145 (43.4)	189 (56.6)	0.208
FD15	1 (20.0)	4 (80.0)	13 (22.4)	45 (77.6)	42 (27.3)	112 (72.7)	84 (25.1)	250 (74.9)	0.886
Total	5 (100.0)		58 (100.0)		154 (100.0)		334 (100.0)		

Pearson Chi-squared test; ** *p* < 0.01 highly statistically significant.

**Table 6:** Features preferred by students in instructional videos—comparative study on preferred internet services: entertainment.

	Main Activity on Internet: Entertainment								*p*-Value
	The Least Important		Less Important		Important		The Most Important		
	Option 1: *n* (%)	Option 2: *n* (%)	Option 1: *n* (%)	Option 2: *n* (%)	Option 1: *n* (%)	Option 2: *n* (%)	Option 1: *n* (%)	Option 2: *n* (%)	
FD1	-	34 (100.0)	1 (0.7)	143 (99.3)	6 (3.2)	184 (96.8)	2 (1.1)	181 (98.9)	0.218
FD2	23 (67.6)	11 (32.4)	70 (48.6)	74 (51.4)	84 (44.2)	106 (55.8)	90 (49.2)	93 (50.8)	0.093
FD3	5 (14.7)	29 (85.3)	21 (14.6)	123 (85.4)	39 (20.5)	151 (79.5)	39 (21.3)	144 (78.7)	0.367
FD4	17 (50.0)	17 (50.0)	47 (32.6)	97 (67.4)	53 (27.9)	137 (72.1)	57 (31.1)	126 (68.9)	0.085
FD5	18 (52.9)	16 (47.1)	103 (71.5)	41 (28.5)	134 (70.5)	56 (29.5)	119 (65.0)	64 (35.0)	0.130
FD6	9 (26.5)	25 (73.5)	36 (25.0)	108 (75.0)	53 (27.9)	137 (72.1)	59 (32.2)	124 (67.8)	0.527
FD7	13 (38.2)	21 (61.8)	71 (49.3)	73 (50.7)	89 (46.8)	101 (53.2)	82 (44.8)	101 (55.2)	0.660
FD8	15 (44.1)	19 (55.9)	44 (30.6)	100 (69.4)	49 (25.8)	141 (74.2)	40 (21.9)	143 (78.1)	0.035 *
FD9	26 (76.5)	8 (23.5)	110 (76.4)	34 (23.6)	143 (75.3)	47 (24.7)	150 (82.0)	33 (18.0)	0.431
FD10	18 (52.9)	16 (47.1)	37 (25.7)	107 (74.3)	65 (34.2)	125 (65.8)	60 (32.8)	123 (67.2)	0.021 *
FD11	8 (23.5)	26 (76.5)	10 (6.9)	134 (93.1)	16 (8.4)	174 (91.6)	18 (9.8)	165 (90.2)	0.027 *
FD12	2 (5.9)	32 (94.1)	4 (2.8)	140 (97.2)	7 (3.7)	183 (96.3)	12 (6.6)	171 (93.4)	0.360
FD13	10 (29.4)	24 (70.6)	12 (8.3)	132 (91.7)	19 (10.0)	171 (90.0)	23 (12.6)	160 (87.4)	0.005 **
FD14	14 (41.2)	20 (58.8)	65 (45.1)	79 (54.9)	97 (51.1)	93 (48.9)	83 (45.4)	100 (54.6)	0.549
FD15	9 (26.5)	25 (73.5)	37 (25.7)	107 (74.3)	47 (24.7)	143 (75.3)	47 (25.7)	136 (74.3)	0.994
Total	34 (100.0)		144 (100.0)		190 (100.0)		183 (100.0)		

Pearson Chi-squared test; * *p* < 0.05 statistically significant; and ** *p* < 0.01 highly statistically significant.

**Table 7:** Features preferred by students in instructional videos—comparative study on preferred internet services: domestic facilities.

	Main Activity on Internet: Domestic Facilities								*p*-Value
	The Least Important		Less Important		Important		The Most Important		
	Option 1: *n* (%)	Option 2: *n* (%)	Option 1: *n* (%)	Option 2: *n* (%)	Option 1: *n* (%)	Option 2: *n* (%)	Option 1: *n* (%)	Option 2: *n* (%)	
FD1	6 (2.1)	278 (97.9)	1 (0.7)	142 (99.3)	1 (1.1)	88 (98.9)	1 (2.9)	34 (97.1)	0.647
FD2	127 (44.7)	157 (55.3)	68 (47.6)	75 (52.4)	55 (61.8)	34 (38.2)	17 (48.6)	18 (51.4)	0.046 *
FD3	60 (21.1)	224 (78.9)	26 (18.2)	117 (81.8)	9 (10.1)	80 (89.9)	9 (25.7)	26 (74.3)	0.089
FD4	88 (31.0)	196 (69.0)	49 (34.3)	94 (65.7)	28 (31.5)	61 (68.5)	9 (25.7)	26 (74.3)	0.781
FD5	202 (71.1)	82 (28.9)	91 (63.6)	52 (36.4)	58 (65.2)	31 (34.8)	23 (65.7)	12 (34.3)	0.403
FD6	85 (29.9)	199 (70.1)	42 (29.4)	101 (70.6)	18 (20.2)	71 (79.8)	12 (34.3)	23 (65.7)	0.272
FD7	129 (45.4)	155 (54.6)	64 (44.8)	79 (55.2)	44 (49.4)	45 (50.6)	18 (51.4)	17 (48.6)	0.814
FD8	79 (27.8)	205 (72.2)	43 (30.1)	100 (69.9)	17 (19.1)	72 (80.9)	9 (25.7)	26 (74.3)	0.304
FD9	225 (79.2)	59 (20.8)	114 (79.7)	29 (20.3)	65 (73.0)	24 (27.0)	25 (71.4)	10 (28.6)	0.451
FD10	82 (28.9)	202 (71.1)	53 (37.1)	90 (62.9)	30 (33.7)	59 (66.3)	15 (42.9)	20 (57.1)	0.186
FD11	21 (7.4)	263 (92.6)	17 (11.9)	126 (88.1)	11 (12.4)	78 (87.6)	3 (8.6)	32 (91.4)	0.346
FD12	12 (4.2)	272 (95.8)	7 (4.9)	136 (95.1)	5 (5.6)	84 (94.4)	1 (2.9)	34 (97.1)	0.902
FD13	27 (9.5)	257 (90.5)	22 (15.4)	121 (84.6)	12 (13.5)	77 (86.5)	3 (8.6)	32 (91.4)	0.281
FD14	139 (48.9)	145 (51.1)	63 (44.1)	80 (55.9)	41 (46.1)	48 (53.9)	16 (45.7)	19 (54.3)	0.805
FD15	71 (25.0)	213 (75.0)	39 (27.3)	104 (72.7)	17 (19.1)	72 (80.9)	13 (37.1)	22 (62.9)	0.195
Total	284 (100.0)		143 (100.0)		89 (100.0)		35 (100.0)		

Pearson Chi-squared test; * *p* < 0.05 statistically significant.

**Table 8:** The description of the clusters identified among the preferred features of didactic videos.

	Identified Clusters					
	Cluster 1 (195 Cases—35.4%)		Cluster 2 (192 Cases—34.8%)		Cluster 3 (164 Cases—29.8%)	
	Option 1: *n* (%)	Option 2: *n* (%)	Option 1: *n* (%)	Option 2: *n* (%)	Option 1: *n* (%)	Option 2: *n* (%)
FD1	2 (1.0)	193 (99.0)	2 (1.0)	190 (99.0)	5 (3.0)	159 (97.0)
FD2	116 (59.5)	79 (40.5)	66 (34.4)	126 (65.6)	85 (51.8)	79(48.2)
FD3	11 (5.6)	184 (94.4)	53 (27.6)	139 (72.4)	40 (24.4)	124 (75.6)
FD4	40 (20.5)	155 (79.5)	48 (25.0)	144 (75.0)	86 (52.4)	78 (47.6)
FD5	186 (95.4)	9 (4.6)	126 (65.6)	66 (34.4)	62 (37.8)	102 (62.2)
FD6	77 (39.5)	118 (60.5)	48 (25.0)	144 (75.0)	32 (19.5)	132 (80.5)
FD7	176 (90.3)	19 (9.7)	23 (12.0)	169 (88.0)	56 (34.1)	108 (65.9)
FD8	47 (24.1)	148 (75.9)	26 (13.5)	166 (86.5)	75 (45.7)	89 (54.3)
FD9	147 (75.4)	48 (24.6)	172 (89.6)	20 (10.4)	110 (67.1)	54 (32.9)
FD10	45 (23.1)	150 (76.9)	10 (5.2)	182 (94.8)	125 (76.2)	39 (23.8)
FD11	-	195 (100.0)	12 (6.3)	180 (93.8)	40 (24.4)	124 (75.6)
FD12	-	195 (100.0)	5 (2.6)	187 (97.4)	20 (12.2)	144 (87.8)
FD13	6 (3.1)	189 (96.9)	8 (4.2)	184 (95.8)	50 (30.5)	114 (69.5)
FD14	104 (53.3)	91 (46.7)	125 (65.1)	67 (34.9)	30 (18.3)	134 (81.7)
FD15	85 (43.6)	110 (56.4)	12 (6.3)	180 (93.8)	43 (26.2)	121 (73.8)

**Table 9:** The comparative study of the clusters identified among the preferred features of instructional videos.

		Cluster 1— FD7 = 1 and FD10 = 2 *n* (%)	Cluster 2— FD7 = 2 and FD10 = 2 *n* (%)	Cluster 3— FD7 = 2 and FD10 = 1 *n* (%)	*p*-Value
Gender					0.941
	Male	48 (24.6)	45 (23.4)	38 (23.2)	
	Female	147 (75.4)	147 (76.6)	126 (76.8)	
Age group					0.147
	18–20 years	100 (51.3)	100 (52.1)	97 (59.1)	
	21–24 years	65 (33.3)	58 (30.2)	35 (21.3)	
	Over 25 years	30 (15.4)	34 (17.7)	32 (19.5)	
Main activity on internet: information (on a scale from 1 to 4)					0.203
	1—the least important	5 (2.6)	6 (3.1)	8 (4.9)	
	2—less important	54 (27.7)	55 (28.6)	29 (17.7)	
	3—important	78 (40.0)	69 (35.9)	67 (40.9)	
	4—the most important	58 (29.7)	62 (32.3)	60 (36.6)	
Main activity on internet: communication (on a scale from 1 to 4)					0.201
	1—the least important	1 (0.5)	3 (1.6)	1 (0.6)	
	2—less important	17 (8.7)	28 (14.6)	13 (7.9)	
	3—important	51 (26.2)	57 (29.7)	46 (28.0)	
	4—the most important	126 (64.6)	104 (54.2)	104 (63.4)	
Main activity on internet: entertainment (on a scale from 1 to 4)					0.119
	1—the least important	8 (4.1)	8 (4.2)	18 (11.0)	
	2—less important	54 (27.7)	52 (27.1)	38 (23.2)	
	3—important	69 (35.4)	69 (35.9)	52 (31.7)	
	4—the most important	64 (32.8)	63 (32.8)	56 (34.1)	
Main activity on internet: domestic facilities (on a scale from 1 to 4)					0.296
	1—the least important	99 (50.8)	112 (58.3)	73 (44.5)	
	2—less important	51 (26.2)	43 (22.4)	49 (29.9)	
	3—important	31 (15.9)	28 (14.6)	30 (18.3)	
	4—the most important	14 (7.2)	9 (4.7)	12 (7.3)	
Total		195 (100.0)	192 (100.0)	164 (100.0)	

## Data Availability

The data presented in this study are available on request from the corresponding author. The data are not publicly available due to ethical and privacy restrictions.

## Appendix A

THE QUESTIONNAIRE’S STRUCTURE:

Please check the features you prefer to find at the instructional videos used for preparation at the specialty objects:

	**Option 1:**	**Option 2:**
FD1: The narrator’s voice is:	☐ Computer generated	☐ Human
FD2: The narrator’s gender is:	☐ Male	☐ Female
FD3: Narrator speaks in:	☐ English	☐ Romanian
FD4: The speed at which the narrator speaks is:	☐ High	☐ Low
FD5: Narrator’s speaking style is:	☐ Conversational, friendly	☐ Formal, didactic
FD6: Soundtrack of the video:	☐ It has background music	☐ It doesn’t have background music
FD7: Visually appealing:	☐ The video contains images for aesthetic purposes only, to fill in the gaps	☐ The video fails to contain images used only for aesthetic purposes, to fill in the gaps
FD8: The narrator’s spoken text is:	☐ Without written text (subtitle) at the bottom of the screen	☐ Accompanied by written text (subtitle) at the bottom of the screen
FD9: Explanatory text accompanying the diagrams and drawings are placed:	☐ Directly on the drawing, in the area they refer to	☐ At the bottom of the screen
FD10: Explanatory diagrams and charts:	☐ They are already drawn, in the videos they are only explained	☐ They are drawn simultaneously with the explanations
FD11: Narrator’s spoken text is:	☐ Without animated diagrams	☐ Accompanied by animated diagrams to explain it better
FD12: Narrator’s spoken text is:	☐ Without graphic, visual elements to mark important notions	☐ Accompanied by graphic, visual elements to mark the important notions
FD13: The instructional video presents the suggested topic:	☐ Heterogenous, as free speech	☐ Structured, explaining first the simple and then complex notions
FD14: The instructional video is designed as:	☐ Regular class—the teacher is recorded at the blackboard, where he explains and draws	☐ PowerPoint presentation with images, diagrams and animated drawings
FD15: The instructional video has a total length of:	☐ Maximum 6 min	☐ Its length is not important

The main activities for which I use Internet are (order them from 1 to 4, according to their importance—1 = the less important; 4 = the most important):	
☐	Information
☐	Communication (e-mail, instant messaging, chatting with friends, dating)
☐	Entertainment (e-books, music, movies, games)
☐	Domestic facilities (shopping online, bills payment, job offers, services offers)
